# Galenic Preparations of Therapeutic *Cannabis sativa* Differ in Cannabinoids Concentration: A Quantitative Analysis of Variability and Possible Clinical Implications

**DOI:** 10.3389/fphar.2018.01543

**Published:** 2019-01-17

**Authors:** Alessandra Bettiol, Niccolò Lombardi, Giada Crescioli, Valentina Maggini, Eugenia Gallo, Alessandro Mugelli, Fabio Firenzuoli, Roberto Baronti, Alfredo Vannacci

**Affiliations:** ^1^Section of Pharmacology and Toxicology, Department of Neurosciences, Psychology, Drug Research and Child Health, University of Florence, Florence, Italy; ^2^Tuscan Regional Centre of Pharmacovigilance and Phytovigilance, Florence, Italy; ^3^Department of Experimental and Clinical Medicine, University of Florence, Florence, Italy; ^4^Center for Integrative Medicine, Careggi University Hospital, University of Florence, Florence, Italy; ^5^Clinical Toxicology Laboratory, Local Health Service, Florence, Italy

**Keywords:** cannabis, galenic preparations, Bediol^®^, Bedrocan^®^, Bedrolite^®^, FM-2^®^, concentrations variability

## Abstract

**Introduction:** Magistral preparations of therapeutic cannabis are extracted from standardized products imported from Holland or from the Florence Military Pharmaceutical Chemical Works, but extraction protocols differ among galenic laboratories. This study assessed the inter-laboratory variability in concentrations of cannabidiol (CBD), cannabinol (CBN), tetrahydrocannabinol (THC), and tetrahydrocannabinolic acid (THCA) among different magistral oil preparations.

**Methods:** 219 samples of Bediol, Bedrobinol, Bedrolite or FM-2 70 or 100 mg/ml in oil were collected from 3 laboratories. Concentrations of CBD, CBN, THC, and THCA were quantified by high-pressure liquid chromatography; inter-laboratories variability was assessed using the Kruskal–Wallis test.

**Results:** A significant variability in CBD and THC concentrations was found for Bediol 70 mg/ml samples from 2 laboratories [for CBD: median 5.4 (range 4.8–6.6) vs. 6.1 (4.9–7.2) mg/ml, *p* = 0.033; for THC: 3.6 (3.1–3.9) vs. 4.0 (2.6–5.1) mg/ml, *p* = 0.020]. As for Bediol 100 mg/ml, a significant variability emerged in THC concentrations among the three considered laboratories [5.7 (-) vs. 4.2 (1.5–4.8) vs. 5.2 (4.2–6.9), *p* = 0.030]. No significant inter-laboratory variability emerged for Bedrocan and Bedrolite. Concentrations of CBD, CBN, and THC were <LOQ in all Bedrocan samples, and CBN and THCA were <LOQ in all Bedrolite samples. As for FM-2, a significant inter-laboratories variability was found for CBD concentrations.

**Conclusion:** Quantitative variability of cannabinoids in magistral preparations might impact on the efficacy and safety of therapeutic cannabis. A standardized protocol is needed to guarantee a homogeneous product and patients’ therapeutic continuity.

## Introduction

In Italy, use of *Cannabis sativa* for Therapeutic Purposes (CTP) was first authorized in 2006. Indications for its use include chronic pain, nausea and vomit associated to chemotherapy, appetite stimulation, hypotension effect in glaucoma, and reduction of uncontrolled body and facial movements (Ministero, 2016).

The main constituent of raw cannabis is tetrahydrocannabinolic acid (THCA) (Moreno-Sanz, 2016). Following heating, THCA is converted to Δ9-THC, a partial agonist on both cannabinoid receptors (CB1 and CB2), that mediates the well-known psychoactive effects of cannabis (Howlett and Abood, 2017). Another active principle, cannabidiol (CBD), has only a modest affinity for the cannabinoid receptors, thus has no psychoactive effects. However, both THC and CBD have a number of additional pharmacological targets, including the calcitonin gene-related peptide (GPR55) orphan receptor, the ligand-gated ion channels of human 5-HT3A receptors, and several additional ionic channels and enzymes (Pertwee, 2008; De Petrocellis et al., 2017).

Magistral preparations of CTP are prepared by extraction from standardized products, obtained from dried and minced cannabis inflorescences and containing standardized THC and CBD concentrations, that are imported in Italy from the Dutch Office of Medicinal cannabis (Ministero, 2016): Bedrocan^®^(mean amounts of 22% for THC and <1% for CBD), Bedrobinol^®^(13.5% for THC and <1% for CBD), Bediol^®^(6.5% for THC and 8% for CBD), and Bedrolite^®^(0.4% THC and 9% CBD) (Office of Medical Cannabis, 2018).

In addition, since 2016 a national production of the cannabis product FM-2^®^has been started in the Military Pharmaceutical Chemical Works of Florence. In the FM-2^®^product, mean amounts of THC and CBD range between 5–8% and 7.5–12%, respectively (Ministero, 2016).

According to the European Pharmacopeia, several magistral products can be prepared, including cannabis decoction filter bags, unit dose formulation for inhalation and cannabis extracts, mainly in olive oil (Council of Europe, 2017).

Although the preparation of cannabis oil is relatively simple (Romano and Hazekamp, 2013), the quali-quantitative composition of the final products is poorly characterized, particularly considering that variations in temperature and time of extraction may impact on the final concentration of cannabinoids (Citti et al., 2016; Pacifici et al., 2017).

Recently, a study conducted on over two hundred extracts highlighted a wide variability in THC and CBD concentrations among different preparations of Bedrocan^®^, Bediol^®^, and Bedrolite^®^5 g/50 ml in olive oil, both inter- and intra-laboratory (Carcieri et al., 2018). In light of these recent findings, our study aimed to provide additional evidence on the variability in terms of concentrations of cannabinoids in different oil preparations from different Italian laboratories, focusing on oil preparations of Bedrocan^®^, Bediol^®^, Bedrolite^®^, and FM-2^®^70 and 100 mg/ml.

## Materials and Methods

Samples of Bedrocan^®^, Bediol^®^, Bedrolite^®^, and FM-2 70 and 100 mg/ml prepared by three different community pharmacies (named Lab1-Lab4) of the Metropolitan Area of Florence, Italy, were collected.

Samples were analyzed at the Department of Clinical Toxicology and Antidoping of the Local Health Authority of Florence, Italy.

### Chemicals

Cannabidiol, cannabinol (CBN), THC, and THCA, were purchased from o2si (Charleston, United States);

Acetonitrile (ACN) and tetrahydrofuran (THF) were purchased from Sigma-Aldrich (St. Louis, MO, United States); methanol was purchased from Honeywell (Seelze, Germany).

For buffer solutions, MilliQ water was obtained from the purification system Millipore^®^(Billerica, MA, United States), whereas K2HPO4 e HCl were purchased from Carlo Erba (Milan, Italy).

### Chromatographic Analysis

High pressure liquid chromatographic analysis with Diode-Array Detection (HPLC-DAD) was performed on a Thermo-Fisher Surveyor Plus (Thermo Fisher Scientific, Waltham, MA, United States).

Chromatographic separation was performed on an Agilent Poroshell^®^120 SB-C18 column, (2.1 mm × 150 mm, with particles diameter of 2.7 μm; Agilent Technologies, Santa Clara, CA, United States), combined with a pre-column SB-C18 (2.1 mm × 5 mm, with particles diameter of 2.7 μm; Agilent Technologies, Santa Clara, CA, United States).

Briefly, 4 preparations of KH2PO4 5 mM (Solution A) at pH of 3.45 were prepared. The calibration lines for analytes were prepared from stock solution of 0.4 mg/ml THC in methanol and of 1 mg/ml CBD in methanol, that were further diluted in methanol to the final concentration of 0.1 mg/ml.

Standard solutions were further diluted to obtain six different samples, with concentrations ranging between 0.001 and 0.040 mg/ml.

Conditioning was performed according to the procedure reported in the data sheet of the column, i.e., with the flow of 10–20 volumes at the speed of 0.4 ml/min over 26 min.

The adopted work conditions were: mobile phases A, ACN/5 mM phosphate butter pH 3.45 at the rate of 75/25 v/v; flow, 0.38 ml/min, temperature of 53°C; injection volume 10 μl full loop; pressure ∼ 210 bar; detector UV channel 222 nm; time of 8 min (isocratic).

### Sample Preparation

Samples were stored at room temperature, until time of analysis.

Forty microliter of the sample were diluted in 960 μl THF. After Vortex mixing, 50 μl of such solution were added to 950 μl of ACN, and the solution was mixed using the Vortex. 10 μl of this solution were injected in the chromatographic system.

### Software

Obtained chromatographs were analyzed using the software ChromQuest^TM^, version 4.2.34 (Thermo Fisher Scientific).

### Statistical Analysis

Concentrations of different active principles in analyzed samples were expressed both as mean value and related standard deviation (SD) and as median value and range (min–max). For the statistical analysis, the value 0.9 mg/ml was arbitrarily attributed to concentrations below the limit of quantification (<LOQ; i.e., <1 mg/ml).

Differences in mean and median concentrations among different pharmacies were tested using the One-way ANOVA test and the Kruskal–Wallis test, respectively.

Statistical analysis was performed using the Software STATA version 14. Statistical significance was considered for *p*-value < 0.05.

## Results

A total of 219 cannabis oil samples was collected from 3 different galenic laboratories. Of them 23 were of Bediol 70 mg/ml, 21 of Bediol 100 mg/ml, 39 of Bedrocan 70 mg/ml, 62 of Bedrocan 100 mg/ml, 14 of Bedrolite 70 mg/ml, 5 of Bedrolite 100 mg/ml, 46 of FM-2 70 mg/ml, and 9 of FM-2 100 mg/ml.

Table 1 summarized the distribution of analyzed cannabis magistral preparations, divided by pharmacy, cannabis strain and concentration.

**Table 1:** Analyzed magistral preparations, divided by laboratory and cannabis strain and concentration.

	No. Bediol		No. Bedrocan		No. Bedrolite		No. FM-2	
	70 mg/ml	100 mg/ml	70 mg/ml	100 mg/ml	70 mg/ml	100 mg/ml	70 mg/ml	100 mg/ml
**Lab 1**	8	1	19	7	5	2	27	6
**Lab 2**		16	1	50		1		3
**Lab 3**	15	4	19	5	9	2	19	
**Tot**	23	21	39	62	14	5	46	9


Concentrations of CBD, CBN, THC, and THA in analyzed cannabis olive oil preparations of Bediol, Bedrocan, Bedrolite, and FM-2 are summarized in Table 2 and expressed as mean value and SD, and as median value and related range. Median value and related interquartile range and range are further illustrated in the Supplementary Figures S1, S2.

**Table 2:** Concentrations of cannabidiol (CBD), cannabinol (CBN), tetrahydrocannabinol (THC) and tetrahydrocannabinolic acid (THCA) in cannabis olive oil preparations of Bediol, Bedrocan, Bedrolite, and FM-2 at 70 and 100 mg/ml, and *p*-value of variability among laboratories from test.

	Overall		Laboratory 1		Laboratory 2		Laboratory 3			
			
	Mean (*SD*)	Median (minimum–maximum)	Mean (*SD*)	Median (IQR; minimum–maximum)	Mean (*SD*)	Median (IQR; minimum–maximum)	Mean (*SD*)	Median (IQR; minimum–maximum)	*F* test statistic; *p*-value from one way ANOVA	*p*-value from Wilcoxon test
**Bediol 70 mg/ml**										
*CBD*	5.9 (0.8)	5.6 (4.8 – 7.2)	5.4 (0.6)	5.4 (4.8 – 6.6)			6.2 (0.8)	6.1 (4.9 – 7.2)	215.96 (<0.001)^∗^	0.033^∗^
*CBN*	<LOQ (0)	<LOQ (<LOQ–<LOQ)	<LOQ (0)	<LOQ (<LOQ–<LOQ)			<LOQ (0)	<LOQ (<LOQ–<LOQ)	–75.67 (1.000)	1.000
*THC*	3.9 (0.6)	3.7 (2.6 – 5.1)	3.6 (0.2)	3.6 (3.1 – 3.9)			4.1 (0.7)	4.0 (2.6 – 5.1)	16.22 (<0.001)^∗^	0.020^∗^
*THCA*	<LOQ (0.1)	<LOQ (<LOQ-1.2)	<LOQ (0)	<LOQ (<LOQ–<LOQ)			<LOQ (0.1)	<LOQ (<LOQ-1.2)	1.07 (0.365)	0.606
**Bediol 100 mg/ml**										
*CBD*	7.2 (1.4)	7.5 (2.8 – 10.5)	8 (–)	8 (–)	6.9 (1.3)	7.4 (2.8 – 8.3)	8.2 (1.7)	8.0 (6.3 – 10.5)	1.42 (0.268)	0.270
*CBN*	<LOQ (0)	<LOQ (<LOQ–<LOQ)	<LOQ (–)	<LOQ (–)	<LOQ (0)	<LOQ (<LOQ–<LOQ)	<LOQ (0)	<LOQ (<LOQ–<LOQ)	–	1.000
*THC*	4.3 (1.0)	4.3 (1.5 – 6.9)	5.7 (–)	5.7 (–)	4.0 (0.7)	4.2 (1.5 – 4.8)	5.4 (1.2)	5.2 (4.2 – 6.9)	5.74 (0.012)^∗^	0.030^∗^
*THCA*	<LOQ (0.1)	<LOQ (<LOQ-1.2)	<LOQ (–)	<LOQ (–)	<LOQ (0.1)	<LOQ (<LOQ-1.2)	1.0 (0.1)	<LOQ (<LOQ-1.2)	0.26 (0.776)	0.903
**Bedrocan 70 mg/ml**										
*CBD*	<LOQ (0.0)	<LOQ (<LOQ – 1.4)	<LOQ (0.0)	<LOQ (<LOQ –<LOQ)	<LOQ (–)	<LOQ (–)	<LOQ (0.1)	<LOQ (<LOQ – 1.4)	0.51 (0.603)	0.961
*CBN*	<LOQ (0.0)	<LOQ (<LOQ –<LOQ)	<LOQ (0.0)	<LOQ (<LOQ –<LOQ)	<LOQ (–)	<LOQ (–)	<LOQ (0.0)	<LOQ (<LOQ –<LOQ)	–	1.000
*THC*	14.0 (2.0)	13.9 (6.1 – 20.0)	13.0 (1.9)	13.5 (6.1 – 14.8)	12 (–)	12 (–)	15.0 (1.7)	15.1 (12.2 – 20.0)	6.64 (0.004)^∗^	0.001^∗^
*THCA*	1.0 (0.4)	<LOQ (<LOQ – 3.2)	1.1 (0.6)	<LOQ (<LOQ – 3.2)	<LOQ (–)	<LOQ (–)	<LOQ (0.0)	<LOQ (<LOQ – <LOQ)	0.89 (0.418)	0.854
**Bedrocan 100 mg/ml**										
*CBD*	<LOQ (0.1)	<LOQ (<LOQ – 1.3)	<LOQ (0.0)	<LOQ (<LOQ –<LOQ)	<LOQ (0.1)	<LOQ (<LOQ – 1.3)	<LOQ (0.0)	<LOQ (<LOQ –<LOQ)	0.82 (0.447)	0.756
*CBN*	<LOQ (0.0)	<LOQ (<LOQ –<LOQ)	<LOQ (0.0)	<LOQ (<LOQ –<LOQ)	<LOQ (0.0)	<LOQ (<LOQ –<LOQ)	<LOQ (0.0)	<LOQ (<LOQ –<LOQ)	–	1.000
*THC*	16.5 (2.4)	16.9 (8.3 – 20.2)	17.1 (3.1)	19.3 (13.1 – 19.7)	16.2 (2.2)	16.8 (8.3 – 19.2)	18.6 (1.9)	19.5 (16.2 – 20.2)	2.66 (0.078)	0.078
*THCA*	<LOQ (<LOQ)	<LOQ (<LOQ – 7.6)	1.9 (2.5)	<LOQ (<LOQ – 7.6)	<LOQ (0.0)	<LOQ (<LOQ – <LOQ)	<LOQ (0.0)	<LOQ (<LOQ – <LOQ)	4.36 (0.017)^∗^	0.829
**Bedrolite 70 mg/ml**										
*CBD*	5.3 (1.3)	5.7 (1.9 – 6.9)	4.7 (1.7)	4.9 (1.9 – 6.2)			5.6 (1.1)	5.9 (3.5 – 6.9)	1.72/(0.215)	0.230
*CBN*	<LOQ (0.0)	<LOQ (<LOQ –<LOQ)	<LOQ (0.0)	<LOQ (<LOQ –<LOQ)			<LOQ (0.0)	<LOQ (<LOQ –<LOQ)	–12.00 (1.000)	1.000
*THC*	<LOQ (0.0)	<LOQ (<LOQ –<LOQ)	<LOQ (0.0)	<LOQ (<LOQ –<LOQ)			<LOQ (0.0)	<LOQ (<LOQ –<LOQ)	–12.00 (1.000)	1.000
*THCA*	<LOQ (0.0)	<LOQ (<LOQ –<LOQ)	<LOQ (0.0)	<LOQ (<LOQ –<LOQ)			<LOQ (0.0)	<LOQ (<LOQ –<LOQ)	–12.00 (1.000)	1.000
**Bedrolite 100 mg/ml**												
*CBD*	6.4 (4.3)	8.6 (<LOQ – 10.2)	5.7 (4.2)	5.7 (2.7 – 8.6)	<LOQ (–)	<LOQ (–)	10.0 (0.3)	10.0 (9.8 – 10.2)	3.28 (0.234)	0.165
*CBN*	<LOQ (0.0)	<LOQ (<LOQ –<LOQ)	<LOQ (0.0)	<LOQ (<LOQ –<LOQ)	<LOQ (–)	<LOQ (–)	<LOQ (0.0)	<LOQ (<LOQ –<LOQ)	–	1.000
*THC*	3.1 (5.0)	<LOQ (<LOQ – 12.0)	<LOQ (0.0)	<LOQ (<LOQ – <LOQ)	12.0 (–)	12.0 (–)	<LOQ (0.0)	<LOQ (<LOQ –<LOQ)	–	0.368
*THCA*	<LOQ (0.0)	<LOQ (<LOQ –<LOQ)	<LOQ (0.0)	<LOQ (<LOQ –<LOQ)	<LOQ (–)	<LOQ (–)	<LOQ (0.0)	<LOQ (<LOQ –<LOQ)	–	1.000
**FM-2 70 mg/ml**										
*CBD*	6.4 (1.1)	6.5 (2.7 – 8.2)	6.1 (1.0)	6.2 (2.7 – 7.2)			6.9 (1.1)	7.0 (3.3 – 8.2)	7.50 (0.009)^∗^	0.001^∗^
*CBN*	<LOQ (0.0)	<LOQ (<LOQ –<LOQ)	<LOQ (0.0)	<LOQ (<LOQ –<LOQ)			<LOQ (0.0)	<LOQ (<LOQ –<LOQ)	–	1.000
*THC*	3.6 (0.6)	3.7 (1.4 – 4.5)	3.6 (0.6)	3.7 (1.4 – 4.2)			3.8 (<LOQ)	3.8 (1.6 – 4.5)	1.21 (0.278)	0.147
*THCA*	<LOQ (0.0)	<LOQ (<LOQ – 1.1)	<LOQ (0)	<LOQ (<LOQ –<LOQ)			<LOQ (0.1)	<LOQ (<LOQ – 1.1)	1.43 (0.237)	0.763
**FM-2 100 mg/ml**										
*CBD*	8.7 (2.1)	9.5 (3.8 – 10.4)	7.9 (2.3)	8.9 (3.8 – 9.8)	10.1 (0.3)	10.1 (9.8 – 10.4)			2.49 (0.159)	0.028^∗^
*CBN*	<LOQ (0.0)	<LOQ (<LOQ –<LOQ)	<LOQ (0.0)	<LOQ (<LOQ –<LOQ)	<LOQ (0.0)	<LOQ (<LOQ –<LOQ)			–	1.000
*THC*	4.7 (1.2)	5.0 (1.7 – 5.8)	4.6 (1.5)	5.2 (1.7 – 5.8)	4.8 (0.3)	4.6 (4.6 – 5.1)			0.03 (0.876)	0.604
*THCA*	<LOQ (0.0)	<LOQ (<LOQ –<LOQ)	<LOQ (0.0)	<LOQ (<LOQ –<LOQ)	<LOQ (0.0)	<LOQ (<LOQ –<LOQ)			–	1.000


*^∗^p-value < 0.05.*

As for Bediol 70 mg/ml, median CBD concentration in the 23 analyzed samples was of 5.6 (4.8–7.2) mg/ml, with a statistically significant variability between the two pharmacies from whom samples were derived (*p*-value from Kruskal–Wallis test = 0.033). As for THC, median concentration was of 3.7 (2.6–5.1) mg/ml, with significant variability between the two pharmacies (*p* = 0.020). Median CBN and THCA concentrations were <LOQ, with no variability between the two pharmacies.

Considering Bediol 100 mg/ml, we found that median THC concentrations significantly differed among the three considered pharmacies: specifically, median THC level was 5.7 (–) in pharmacy 1, 4.2 (1.5–4.8) in pharmacy 2, and 5.2 (4.2–6.9) in pharmacy 3 (*p* = 0.030). Median concentrations of CBD, CBN, and THCA were comparable among samples derived from the three pharmacies.

No statistically significant difference emerged in the concentrations of the active principles CBD, CBN, THC, and THCA among Bedrocan 70 or 100 mg/ml prepared in the three different pharmacies. Of particular note, median levels of CBD, CBN, and THC in Bedrocan samples were <LOQ in all three pharmacies.

Also considering Bedrolite 70 or 100 mg/ml, no difference in the median concentrations of CBD, CBN, THC, and THCA emerged among the three pharmacies. Of note, concentrations of CBN, THC and THCA were <LOQ in all 14 samples of Bedrolite 70 mg/ml, and concentrations of CBN and THCA were <LOQ also in all 5 samples of Bedrolite 100 mg/ml.

Considering FM-2 preparations, we found that median CBD concentrations significantly differed between the pharmacies, both considering FM-2 70 or 100 mg/ml.

Specifically, median CBD level in FM-2 70 mg/ml samples was 6.2 (2.7–7.2) in pharmacy 1 and 7.0 (3.3–8.2) in pharmacy 3 (*p* = 0.001), whereas in FM 100 mg/ml median CBD level was 8.9 (3.8–9.8) in pharmacy 1 and 10.1 (9.8–10.4) in pharmacy 2 (*p* = 0.028). Median concentrations of CBD, CBN, and THCA were comparable among samples derived from the different pharmacies, both considering FM-2 70 and 100 mg/ml.

## Discussion

Increasing literature evidence supports the use of therapeutic cannabis in the treatment of different painful or degenerative clinical conditions (Balash et al., 2017; Neale, 2017; Habib and Artul, 2018; Landa et al., 2018; Lattanzi et al., 2018; Poli et al., 2018), although dosing standards and quality assurance of cannabis-derived medications is still a matter of debate.

Our study highlighted a significant inter-laboratory variability of cannabinoids concentrations among magistral oil preparations. The highest variability in the extraction yields was observed for Bediol^®^-based preparations, both considering CBD and THC concentrations. Significant variability was observed also for CBD concentrations in FM-2 preparations.

Bedrocan^®^-based preparations had the highest extraction yields of THC, whereas CBD, CBN, and THCA were undetectable in these samples; on the other hand, Bedrolite^®^-based concentrations had undetectable concentrations of CBN, THC, and THCA.

These results are in line with a recent study by Carcieri et al. (2018), highlighting a wide inter- and intra-laboratory variability in THC and CBD concentrations in different preparations of Bedrocan, Bediol, and Bedrolite 100 mg/ml. Specifically, authors reported that Bediol^®^-based preparations had significantly higher extraction yields both of THC as compared to Bedrocan^®^and of CBD as compared to Bedrolite^®^.

Our study confirms such variability in concentrations of active principles in Bedrocan, Bediol, and Bedrolite magistral preparations, while adding new evidence on the variability of extraction yields in FM-2 preparation. As extensively described in Carcieri et al. (2018), variability in active pharmaceutical ingredients can be explained by the lack of a unique standardized extraction protocol (Williamson and Evans, 2000; Ware and Tawfik, 2005) as well as by the (although declared) variability in the concentrations of cannabinoids in the imported products (Office of Medical Cannabis, 2018).

Taken together, our results remark the need of introducing a standardized protocol and of providing concentrations data for each preparation; furthermore, these findings open a question on the possible clinical implications of such variability.

A recent study evaluating the effects of different doses of CBD on anxiety in healthy individuals showed that the anxiolytic effect of CBD follows a U-shaped dose-response curve (Linares et al., 2018): personal anxiety was reduced with CBD 300 mg, but not with CBD doses of 100 and 900 mg.

Besides implications on efficacy, dose variability may be associated with safety concerns.

In a phase 1 randomized controlled trial (Ahmed et al., 2014), high THC doses (6.5 mg vs. 3 mg) were associated with significantly higher rates of adverse events. Furthermore, high THC dose has been correlated with deficits in fine motor control and motor timing (Boggs et al., 2018).

Notably, clinical implications of the described heterogeneity in active principles might be particularly relevant in specific population subsets, such as elderly. The elderly is one of most represented population of cannabis users, with an estimated prevalence of use ranging between 6.5 and 37% (Ware and Tawfik, 2005; Hazekamp and Heerdink, 2013; Hazekamp et al., 2013; Ahmed et al., 2014). Considering the well-known pharmacokinetic alternation occurring in such population, the high variability in concentrations of cannabinoids might suggest relevant safety as well as efficacy concerns, highlighting the need of a close phytovigilance monitoring (Lucenteforte et al., 2017).

The major limitation of this study is the small number of analyzed samples and of galenic laboratories from which they were derived. The evaluation of a broader spectrum of samples prepared in different laboratories throughout Italy would be of help to better estimate the real entity of the described variability in cannabinoids extraction yields.

Our results claim the need of a unique defined protocol, to be adopted by all galenic laboratories in order to guarantee reproducible and controlled extraction yields, with the final aim of limiting the possible clinical effects related to over- or under-therapeutic doses. Further studies are needed to evaluated the real correlation between cannabinoids doses and clinical outcomes in the real clinical practice.

## Data Availability

Datasets are available on request.

## Author Contributions

RB, AB, and AV contributed to study design, assisted by NL, GC, VM, EG, AM, and FF. RB and AB took the lead in data analysis, assisted by NL, GC, VM, EG, AM, FF, and AV. GC, FF, NL, AM, and AV performed the data interpretation, assisted by AB, VM, EG, and RB. AB wrote the manuscript, assisted by NL, GC, VM, EG, AM, FF, RB, and AV. EG, VM, and AV revised the manuscript. All authors approved the final version of the manuscript.

## Conflict of Interest Statement

The authors declare that the research was conducted in the absence of any commercial or financial relationships that could be construed as a potential conflict of interest.

## Supplementary Material

FIGURE S1 Distribution of concentrations of cannabidiol (CBD), cannabinol (CBN), tetrahydrocannabinol (THC), and tetrahydrocannabinolic acid (THCA) in cannabis olive oil preparations of Bediol, Bedrocan and Bedrolite 70 and 100 mg/ml from three different laboratories.

FIGURE S2 Distribution of concentrations of CBD, CBN, THC, and THCA in cannabis olive oil preparations of FM-2 70 and 100 mg/ml from three different laboratories.

## References

[B1] Ahmed A. I. A. van den Elsen G. A. H. Colbers A. van der Marck M. A. Burger D. M. Feuth T. B. (2014). Safety and pharmacokinetics of oral delta-9-tetrahydrocannabinol in healthy older subjects: a randomized controlled trial. *Eur. Neuropsychopharmacol.* 24 1475–1482. 10.1016/j.euroneuro.2014.06.007 25035121

[B2] Balash Y. Bar-Lev Schleider L. Korczyn A. D. Shabtai H. Knaani J. Rosenberg A. (2017). Medical cannabis in Parkinson disease: real-life patients’. *Exp. Clin. Neuropharmacol.* 40 268–272. 10.1097/WNF.0000000000000246 29059132

[B3] Boggs D. L. Cortes-Briones J. A. Surti T. Luddy C. Ranganathan M. Cahill J. D. (2018). The dose-dependent psychomotor effects of intravenous delta-9-tetrahydrocannabinol (Δ9-THC) in humans. *J. Psychopharmacol.* 32 1308–1318. 10.1177/0269881118799953 30255720

[B4] Carcieri C. Tomasello C. Simiele M. De Nicolò A. Avataneo V. Canzoneri L. (2018). Cannabinoids concentration variability in cannabis olive oil galenic preparations. *J. Pharm. Pharmacol.* 70 143–149. 10.1111/jphp.12845 29057480

[B5] Citti C. Ciccarella G. Braghiroli D. Parenti C. Vandelli M. A. Cannazza G. (2016). Medicinal cannabis: principal cannabinoids concentration and their stability evaluated by a high performance liquid chromatography coupled to diode array and quadrupole time of flight mass spectrometry method. *J. Pharm. Biomed. Anal.* 128 201–209. 10.1016/j.jpba.2016.05.033 27268223

[B6] Council of Europe (2017). *European Pharmacopoeia*, 9th Edn Strasbourg: EDQM.

[B7] De Petrocellis L. Nabissi M. Santoni G. Ligresti A. (2017). Actions and regulation of ionotropic cannabinoid receptors. *Adv. Pharmacol.* 80 249–289. 10.1016/bs.apha.2017.04.001 28826537

[B8] Habib G. Artul S. (2018). Medical cannabis for the treatment of fibromyalgia. *J. Clin. Rheumatol.* 24 255–258. 10.1097/RHU.0000000000000702 29461346

[B9] Hazekamp A. Heerdink E. R. (2013). The prevalence and incidence of medicinal cannabis on prescription in the Netherlands. *Eur. J. Clin. Pharmacol.* 69 1575–1580. 10.1007/s00228-013-1503-y 23588562

[B10] Hazekamp A. Ware M. A. Muller-Vahl K. R. Abrams D. Grotenhermen F. (2013). The medicinal use of cannabis and cannabinoids—an international cross-sectional survey on administration forms. *J. Psychoactive Drugs* 45 199–210. 10.1080/02791072.2013.805976 24175484

[B11] Howlett A. C. Abood M. E. (2017). CB1 and CB2 receptor pharmacology. *Adv. Pharmacol.* 80 169–206. 10.1016/bs.apha.2017.03.007 28826534 PMC5812699

[B12] Landa L. Jurica J. Sliva J. Pechackova M. Demlova R. (2018). Medical cannabis in the treatment of cancer pain and spastic conditions and options of drug delivery in clinical practice. *Biomed. Pap. Med. Fac. Univ. Palacky. Olomouc. Czech. Repub.* 162 18–25. 10.5507/bp.2018.007 29560966

[B13] Lattanzi S. Brigo F. Cagnetti C. Trinka E. Silvestrini M. (2018). Efficacy and safety of adjunctive cannabidiol in patients with lennox-gastaut syndrome: a systematic review and meta-analysis. *CNS Drugs* 32 905–916. 10.1007/s40263-018-0558-9 30132269

[B14] Linares I. M. Zuardi A. W. Pereira L. C. Queiroz R. H. Mechoulam R. Guimarães F. S. (2018). Cannabidiol presents an inverted U-shaped dose-response curve in a simulated public speaking test. *Braz. J. Psychiatry* 10.1590/1516-4446-2017-0015 [Epub ahead of print]. 30328956 PMC6781714

[B15] Lucenteforte E. Lombardi N. Vetrano D. L. La Carpia D. Mitrova Z. Kirchmayer U. (2017). Inappropriate pharmacological treatment in older adults affected by cardiovascular disease and other chronic comorbidities: a systematic literature review to identify potentially inappropriate prescription indicators. *Clin. Interv. Aging* 12 1761–1778. 10.2147/CIA.S137403 29089750 PMC5656349

[B16] Ministero della Salute (2016). *Uso Medico Della Cannabis.* Available at: http://www.salute.gov.it/portale/temi/p2_6.jsp?lingua=italiano&id=4587&area=sostanzeStupefacenti&menu=vuoto (accessed 10 23 2018).

[B17] Moreno-Sanz G. (2016). Can you pass the acid test? critical review and novel therapeutic perspectives of Δ9-tetrahydrocannabinolic acid A. *Cannabis Cannabinoid Res.* 1 124–130. 10.1089/can.2016.0008 28861488 PMC5549534

[B18] Neale M. (2017). Efficacy and safety of cannabis for treating children with refractory epilepsy. *Nurs. Child. Young People* 29 32–37. 10.7748/ncyp.2017.e907 29115760

[B19] Office of Medical Cannabis (2018). *Medical Cannabis – Types of Medical Cannabis.* Available at: https://www.cannabisbureau.nl/english/medicinal-cannabis (accessed 10 23 2018).

[B20] Pacifici R. Marchei E. Salvatore F. Guandalini L. Busardò F. P. Pichini S. (2017). Evaluation of cannabinoids concentration and stability in standardized preparations of cannabis tea and cannabis oil by ultra-high performance liquid chromatography tandem mass spectrometry. *Clin. Chem. Lab. Med.* 55 1555–1563. 10.1515/cclm-2016-1060 28207408

[B21] Pertwee R. G. (2008). The diverse CB1 and CB2 receptor pharmacology of three plant cannabinoids: delta9-tetrahydrocannabinol, cannabidiol and delta9-tetrahydrocannabivarin. *Br. J. Pharmacol.* 153 199–215. 10.1038/sj.bjp.0707442 17828291 PMC2219532

[B22] Poli P. Crestani F. Salvadori C. Valenti I. Sannino C. (2018). Medical cannabis in patients with chronic pain: effect on pain relief, pain disability, and psychological aspects. A prospective non randomized single arm clinical trial. *Clin. Ter.* 169 e102–e107. 10.7417/T.2018.2062 29938740

[B23] Romano L. L. Hazekamp A. (2013). Cannabis oil: chemical evaluation of an upcoming cannabis-based medicine. *Cannabinoids* 1 1–11.

[B24] Ware M. A. Tawfik V. L. (2005). Safety issues concerning the medical use of cannabis and cannabinoids. *Pain Res. Manag.* 10(Suppl. A), 31A–37A. 10.1155/2005/312357 16237480

[B25] Williamson E. M. Evans F. J. (2000). Cannabinoids in clinical practice. *Drugs* 60 1303–1314. 10.2165/00003495-200060060-00005 11152013

