# Supplementary figures and images for: Galenic Preparations of Therapeutic Cannabis sativa Differ in Cannabinoids Concentration: A Quantitative Analysis of Variability and Possible Clinical Implications
Source: Front Pharmacol. 2019 Jan 17;9:1543. doi: 10.3389/fphar.2018.01543 (PMC6344428; doi:10.3389/fphar.2018.01543)

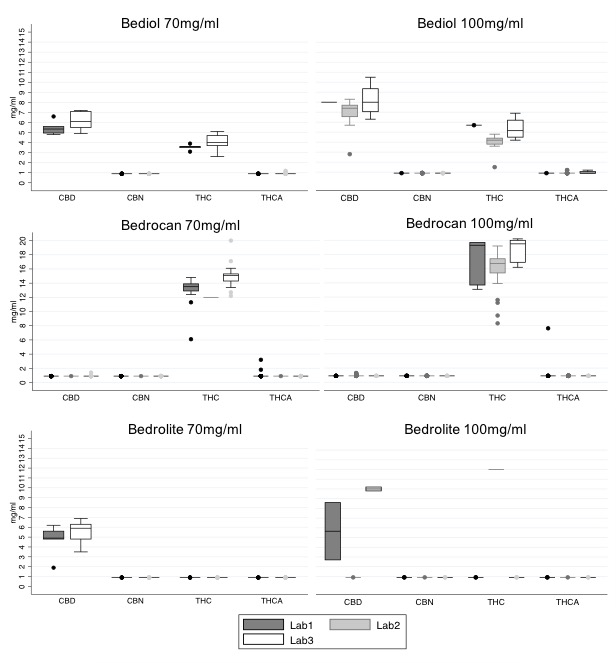

Supplement: FIGURE S1 — Distribution of concentrations of cannabidiol (CBD), cannabinol (CBN), tetrahydrocannabinol (THC), and tetrahydrocannabinolic acid (THCA) in cannabis olive oil preparations of Bediol, Bedrocan and Bedrolite 70 and 100 mg/ml from three different laboratories. [file Image_1.JPEG]

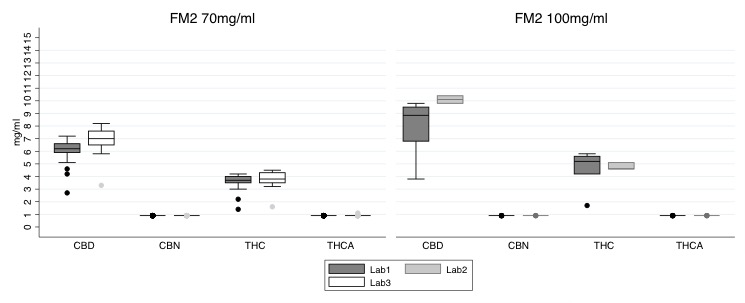

Supplement: FIGURE S2 — Distribution of concentrations of CBD, CBN, THC, and THCA in cannabis olive oil preparations of FM-2 70 and 100 mg/ml from three different laboratories. [file Image_2.JPEG]
